# Effects of Allyl Isothiocyanate on Oxidative and Inflammatory Stress in Type 2 Diabetic Rats

**DOI:** 10.3390/molecules27175568

**Published:** 2022-08-29

**Authors:** Monika Okulicz, Iwona Hertig, Ewelina Król, Tomasz Szkudelski

**Affiliations:** 1 Department of Animal Physiology, Biochemistry and Biostructure, Faculty of Veterinary Medicine and Animal Sciences, Poznań University of Life Sciences, Wołyńska 35, 60-637 Poznań, Poland; 2 Department of Human Nutrition and Dietetics, Poznań University of Life Sciences, Wojska Polskiego 31, 60-624 Poznań, Poland

**Keywords:** allyl isothiocyanate, diabetes, ROS, inflammation, trace elements

## Abstract

Oxidative stress and inflammation play a crucial role in the pathogenesis and progression of diabetes. Currently, there is a growing need to exploit plant-derived bioactive compounds to support conventional therapies. The purpose of this study was to explore allyl isothiocyanate (AITC) potency in reducing oxidative and inflammatory stress along with its profitable modulation trace element status in pathological conditions such as diabetes. Two weeks of oral AITC treatments (2.5, 5, and 25 mg/kg body weight per day) were evaluated in Wistar rats with diabetes induced by a high-fat diet and streptozotocin. The study included AITC influence on antioxidant factors (SOD, CAT, GST, Nrf2), stress and inflammatory markers (cortisol, CRP, IL-1β, IL-6, TNFα, NF-κB), lipid peroxidation indices (TBARS, -SH groups), and trace element status (Fe, Zn, and Cu) in the detoxification and lymphoid organs. Independently of dose, AITC increased cortisol levels in rat blood serum and decreased total thiol groups (T-SH) and protein-bound thiol groups (PB-SH) collaterally with raised thiobarbituric acid reactive substances (TBARS) in diabetic rat liver. The inflammation and oxidative effects were enhanced by an AITC dose increase. The highest dose of AITC, 25 mg/kg b.w., strongly affected the inflammation process by increasing IL-6, IL-1β, and TNFα in the blood serum, and it upregulated Nrf2 transcription factor with increased SOD, GPx, and GST activities in the liver. AITC showed an equivocal effect on profitable modulation of disturbances in mineral homeostasis in the liver, kidney, and spleen. Our findings revealed that two-week AITC treatment exacerbated oxidative and inflammation status in diabetic rats.

## 1. Introduction

Currently, there is increasing demand for natural products in order to improve health and prevent chronic diseases [1,2]. In particular, there is a growing need to exploit plant-derived bioactive compounds with antidiabetic potential to support conventional therapies [3]. This need arises from the fact that long-term consumption of antidiabetic drugs is associated with side effects [4]. One such bioactive compound is the aliphatic and highly volatile allyl isothiocyanate (AITC), a natural compound widely found in many cruciferous vegetables [5]. Numerous preclinical studies demonstrate AITC’s antimicrobial, chemopreventive, and lately, anti-obesity properties due to its detoxification, antioxidant, and anti-inflammatory activity [6,7,8,9]. Diabetes mellitus is a disease with increased mortality and morbidity rates among many populations worldwide due to key factors such as aging, urbanization, obesity, and reduced physical activity. Oxidative stress plays a crucial role in the pathogenesis and progression of diabetes. An increase in oxygen and nitrogen free radicals (ROS/RNS) is associated with enhanced non-enzymatic protein glycation and glucose oxidation [10]. High levels of lipid peroxidation products are a pivotal marker of oxidative stress; proinflammatory cytokines increase, including a drop in non-enzymatic antioxidants such as glutathione (GSH) levels in diabetes [11,12]. Nuclear erythroid 2-related factor 2 (Nrf2) and nuclear factor-κB (NF-κB) are vital regulators in all these processes, with a leading role in oxidative stress and inflammation control. It has been reported that ITCs increase Nrf2 activity and inhibit NF-κB [8,13]. So far, AITC has shown crucial antioxidant properties in plants, where it is used as a natural insect repellent. Ugolini et al. [14] noted that kiwifruit fumigation with AITC triggered an initial oxidative burst and improved its antioxidant potential. Additionally, the effect of *Sitophilus zeamais* insect fumigation with AITC on catalase and copper/zinc superoxide dismutase expression was analyzed by Zhang et al. [15]. The mRNA level of CAT was strongly downregulated, but CuZnSOD expression was increased following exposure to AITC. In the *Arabidopsis thaliana* plant, it was noted that AITC elevated expression of GST-encoding genes [16].

None of the nutrients and enzymes acts in isolation from the others. So, the antioxidant balance disturbance can be caused by excess or deficiency of a trace element such as Fe, and the ratio Cu to Zn, which also results in oxidative stress generation at the cellular and tissue level [17]. Fe, Cu, and Zn play a crucial role in many biochemical redox reactions due to their presence in the catalytic centers of various enzymes [18]. Excessive accumulation of Fe and Cu in the liver and other tissues exacerbates oxidative stress through the Fenton reaction, causing diabetic complications. Additionally, elevated Fe stores are associated with decreased insulin sensitivity (insulin resistance) [19]. On the other hand, Zn is necessary for insulin secretion, insulin receptor binding, and maintenance of antioxidant balance [20]. Moreover, Zn can facilitate insulin-induced glucose transport [18]. An imbalance of the Cu/Zn ratio seems to be a better inflammatory–nutritional biomarker than Zn or Cu status alone. Elevated Cu/Zn ratios are associated with oxidative stress, inflammation, and disrupted immune status [21]. Recently, we have shown that AITC affects hormone levels and metabolic status in diabetic rats [22]. However, there are no sufficient data on the influence of this compound on oxidative and inflammatory stress in rats with experimentally induced diabetes. With this background in mind, this study was designed to explore AITC potency in reducing oxidative and inflammatory stress along with its profitable modulation trace element status in pathological conditions such as diabetes.

## 2. Results

### 2.1. Effects of AITC on Fe, Cu, and Zn Content in the Liver, Kidney, and Spleen of Diabetic Rats

In diabetic rats, the trace elements status was disturbed in the liver. Diabetic rats displayed a substantial rise in Fe (*p* ≤ 0.01) and a drop in Cu content (*p* ≤ 0.01). AITC administration in a dose-dependent manner restored the balance of Fe and Zn in the diabetic rat liver. A total of 2.5 mg/kg b.w. AITC favorably lowered Fe (*p* ≤ 0.05) but adversely dropped Zn (*p* ≤ 0.05) content in the liver. Conversely, a 2-fold higher dose, 5 mg/kg b.w., increased the Fe (*p* ≤ 0.05) liver amount. Moreover, a moderate, not significant rise in Fe amount was also noted in the kidney and spleen after AITC treatment. AITC at two lower doses (2.5; 5.0 mg/kg) lowered Cu (*p* ≤ 0.01 and *p* ≤ 0.05, respectively) only in diabetic rat spleen, with unchanged Cu content in the liver and kidney. So, due to a significant drop in spleen Cu content, a favorable splenic decreased Cu/Zn ratio was observed following 5 mg/kg AITC treatment (*p* ≤ 0.01) (Table 1).

### 2.2. Effects of AITC on Antioxidant Enzyme Activities and Transcription Factor Content in Diabetic Rats

**Figure 1:**
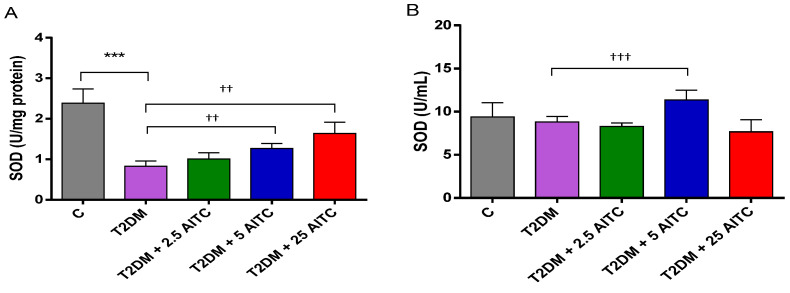

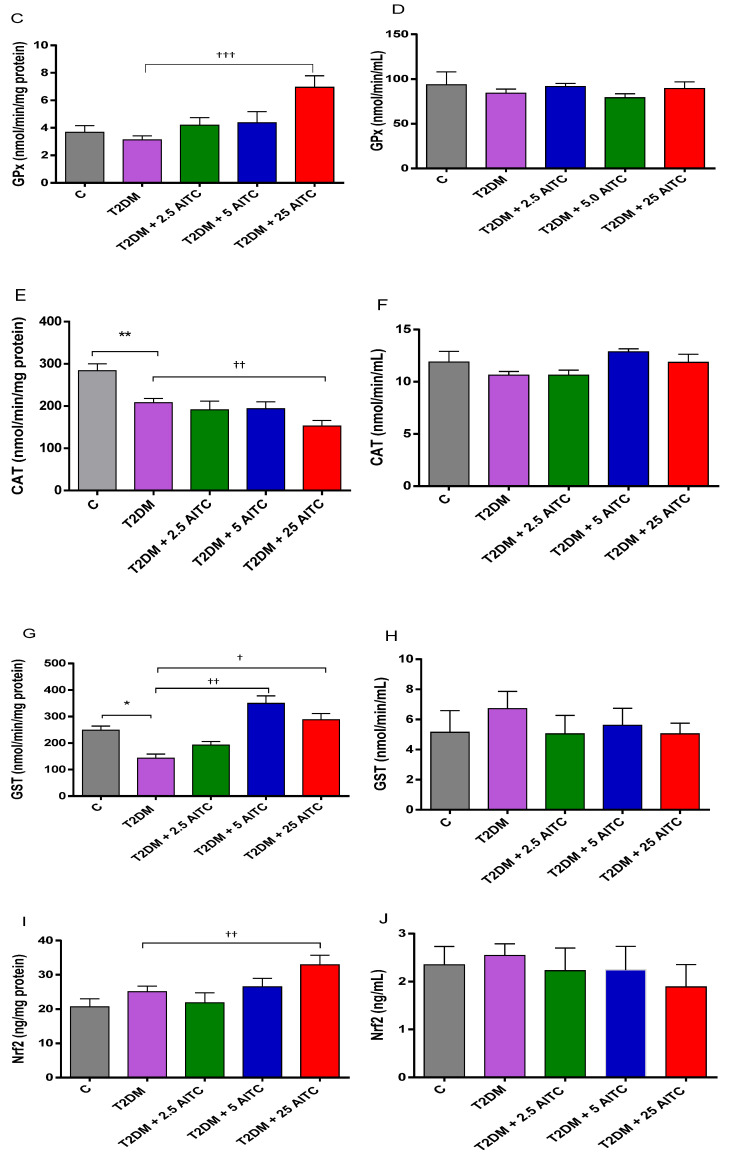
Effects of AITC on antioxidant enzyme activities: SOD (**A**,**B**), GPx (**C**,**D**), CAT (**E**,**F**), GST (**G**,**H**) and Nrf2 transcription factor contents (**I**,**J**) in the liver and the blood serum of diabetic rats. Each column represents the mean ± SEM (*n* = 8). * *p* ≤ 0.05, ** *p* ≤ 0.01, *** *p* ≤ 0.001 compared with C group; † *p* ≤ 0.05, †† *p* ≤ 0.01, ††† *p* ≤ 0.001 compared with T2DM group. Abbreviations: C—control group; T2DM—high-fat diet with STZ-injected group; T2DM +AITC 2.5; AITC 5; AITC 25—high-fat diet with STZ-injected group with oral administration of 2.5; 5; 25 mg/kg b.w. AITC, respectively.

The diabetic rats showed significantly lower liver SOD (*p* ≤ 0.001), CAT (*p* ≤ 0.01), and GST (*p* ≤ 0.05) activities than healthy control animals. Distinctively, the highest dose of AITC 25 mg/kg b.w. caused elevation of SOD (*p* ≤ 0.01), GPx activity (*p* ≤ 0.001) with Nrf2 transcription factor increase (*p* ≤ 0.01), and CAT activity drop (*p* ≤ 0.01) in the liver. AITC at doses 5; 25 mg/kg b.w. increased GST activity (*p* ≤ 0.01; *p* ≤ 0.05, respectively) in the rat liver. There were no considerable changes in enzyme activity and transcription factor Nrf2 concentration in the blood serum except SOD increase (*p* ≤ 0.001) after 5 mg/kg b.w. AITC treatment. Additionally, AITC at this dose enhanced SOD activity (*p* ≤ 0.01) in the liver (Figure 1A–J).

### 2.3. Effects of AITC on Inflammatory Markers in Diabetic Rats

**Figure 2:**
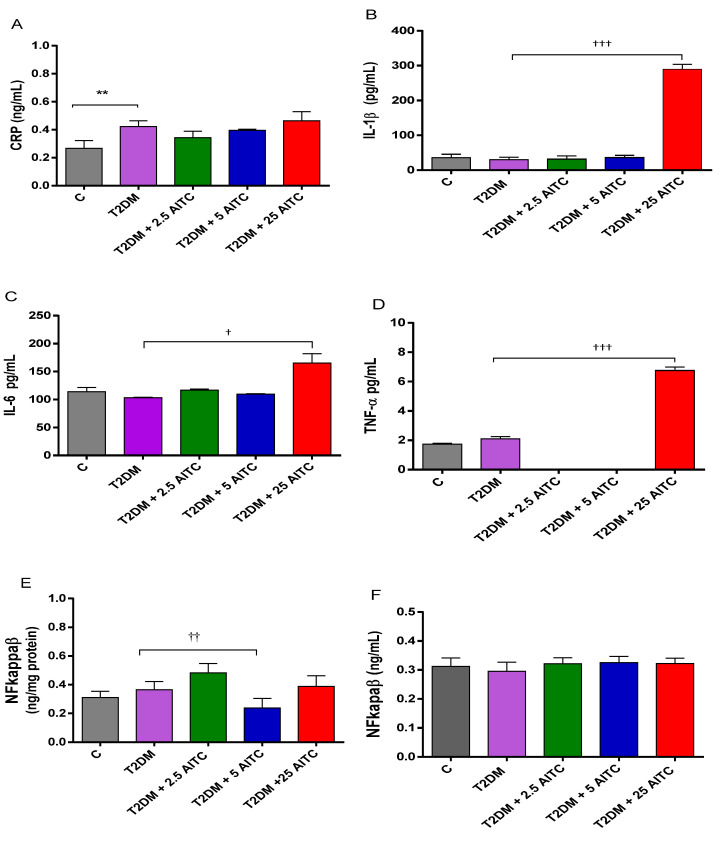
Effects of AITC on inflammatory markers: CRP (**A**), IL-1β (**B**), IL-6 (**C**), TNFα (**D**) concentrations in the blood serum and NF-κB transcription factor contents in the liver (**E**) and the blood serum (**F**) of diabetic rats. Each column represents the mean ± SEM (*n* = 8). ** *p* ≤ 0.01 compared with C group; † *p* ≤ 0.05, †† *p* ≤ 0.01, ††† *p* ≤ 0.001 compared with T2DM group. Abbreviations: C—control group; T2DM—high-fat diet with STZ-injected group; T2DM +AITC 2.5; AITC 5; AITC 25—high-fat diet with STZ-injected group with oral administration of 2.5; 5; 25 mg/kg b.w. AITC, respectively.

Control diabetic rats revealed that symptoms of inflammation by blood serum CRP increased (*p* ≤ 0.01). Notably, the highest dose of AITC 25 mg/kg b.w. strongly affected the inflammation process by increasing biomarkers inflammation cytokine levels IL-1β (*p* ≤ 0.001), IL-6 (*p* ≤ 0.05), and TNFα (*p* ≤ 0.001) in the blood serum. TNFα at 2.5; 5.0 AITC was below the detectable values. Additionally, the medium dose of AITC 5.0 mg/kg b.w. decreased the transcription factor NF-kappaB contents in the diabetic liver (Figure 2A–F).

### 2.4. Effects of AITC on Cortisol Levels in Diabetic Rats

**Figure 3:**
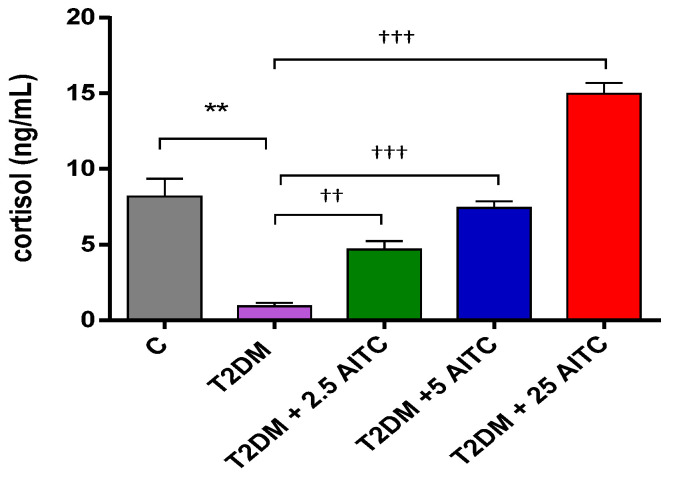
Effects of AITC on cortisol levels in the blood serum of diabetic rats.Each column represents the mean ± SEM (*n* = 8). ** *p* ≤ 0.01 compared with C group; †† *p* ≤ 0.01, ††† *p* ≤ 0.001 compared with T2DM group. Abbreviations: C—control group; T2DM—high-fat diet with STZ-injected group; T2DM +AITC 2.5; AITC 5; AITC 25—high-fat diet with STZ-injected group with oral administration of 2.5; 5; 25 mg/kg b.w. AITC, respectively.

In diabetic rats, the cortisol concentration was considerably decreased (*p* ≤ 0.01). AITC at all doses increased cortisol concentrations in the blood serum (*p* ≤ 0.01; *p* ≤ 0.001; *p* ≤ 0.001, respectively) (Figure 3).

### 2.5. Effects of AITC on Lipid Peroxidation Marker in Diabetic Rats

**Figure 4:**
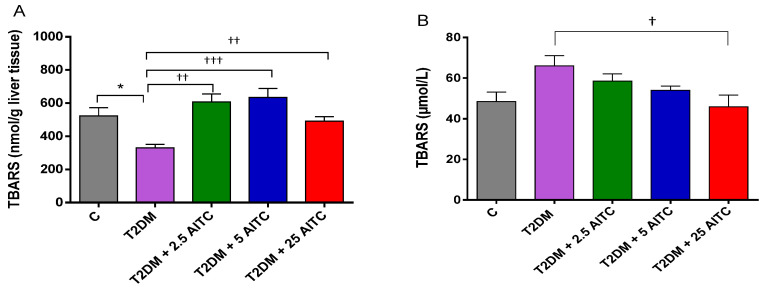
Effects of AITC on thiobarbituric acid reactive substances (TBARS) amount in the liver (**A**) and in the blood serum (**B**) of diabetic rats. Each column represents the mean ± SEM (*n* = 8). * *p* ≤ 0.05 compared with C group; † *p* ≤ 0.05, †† *p* ≤ 0.01, ††† *p* ≤ 0.001 compared with T2DM group. Abbreviations: C—control group; T2DM—high-fat diet with STZ-injected group; T2DM +AITC 2.5; AITC 5; AITC 25—high-fat diet with STZ-injected group with oral administration of 2.5; 5; 25 mg/kg b.w. AITC, respectively.

Surprisingly, thiobarbituric acid reactive substances (TBARS; the standard lipid peroxidation marker) content was lowered in diabetic rats (*p* ≤ 0.05). AITC significantly raised TBARS in the liver independently of the dose (*p* ≤ 0.01; *p* ≤ 0.001; *p*≤ 0.01, respectively). Surprisingly, the highest dose 25 mg/kg b.w. decreased TBARS level in the blood serum (*p*≤ 0.05) (Figure 4A,B).

### 2.6. Effects of AITC on -SH Groups in Diabetic Rats

**Figure 5:**
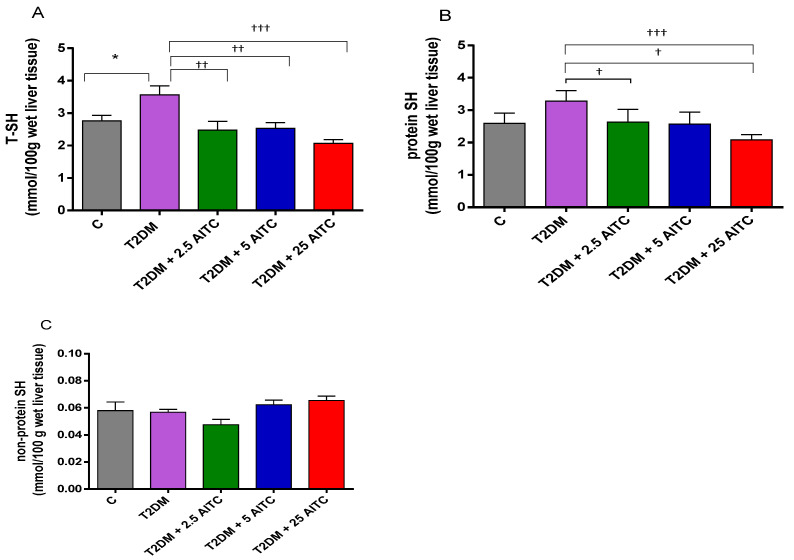
Effects of AITC on total-(T–SH) (**A**), protein-bound (PB–SH) (**B**), and nonprotein (NP–SH) (**C**) sulfhydryl groups content in the liver of diabetic rats. Each column represents the mean ± SEM (*n* = 8). * *p* ≤ 0.05 compared with C group; † *p* ≤ 0.05, †† *p* ≤ 0.01, ††† *p* ≤ 0.001 compared with T2DM group. Abbreviations: C—control group; T2DM—high-fat diet with STZ-injected group; T2DM +AITC 2.5; AITC 5; AITC 25—high-fat diet with STZ-injected group with oral administration of 2.5; 5; 25 mg/kg b.w. AITC, respectively.

Total thiol groups (T-SH) content was increased in diabetic rats (*p* ≤ 0.05). AITC at all doses decreased T-SH (*p* ≤ 0.01; *p* ≤ 0.01; *p* ≤ 0.001, respectively) along with PB-SH amount in the liver (*p* ≤ 0.05; *p* ≤ 0.05; *p* ≤ 0.001, respectively). None of the AITC doses influenced glutathione (NP-SH) content (Figure 5A–C).

## 3. Discussion

So far, numerous research studies have revealed the antioxidative and anti-inflammatory properties of AITC. Ahn [23] showed that 5 and 50 mg/kg b.w. AITC treatment ameliorates oxidative liver injury by suppressing the SOD and CAT reduction and malondialdehyde; lipid peroxidation product (MDA) elevation in ClC_4_-intoxicated rats. Very recently Waz et al. [24] noted that 25 mg/kg/day of AITC administration to adult male albino rats for 7 days alleviated myocardial oxidative stress caused by doxorubicin. AITC attenuated the increase in MDA and nitric oxide (NO) and elevating the antioxidant reduced the glutathione (GSH) level as well as superoxide dismutase (SOD) activity. In our AITC-treated diabetic rats, the increased SOD, GPx, and GST enzyme activities were consistent with the observation mentioned above. However, it was accompanied by a simultaneous decrease in protein-bound -SH groups and raised lipid peroxidation indicators (TBARS).

This would imply the presence of oxidative stress in diabetic rats in the company of AITC. It is commonly known that electrophiles, to which AITC belongs, are highly reactive molecules binding thiols. They participate in the biological oxidation-reduction reactions [25,26]. AITC reacts easily with free amino and thiol groups [27]. In in vitro studies, AITC was shown to form adducts of cysteine-containing proteins or glutathione. In this way, AITC disturbed the intracellular redox balance by protein structure and function modification [14,28]. This leads to the generation of reactive oxygen species (ROS). Previously, such AITC conjugation to protein-bound -SH groups and depletion of GSH have been reported in plant cells [16]. The direct interaction between AITC and protein-bound -SH groups, noted in our study, seemed to contribute to the pro-oxidant effects in diabetic conditions. Interestingly, the GSH pool remained unchanged despite the noted GST activity increase. The lack of glutathione alteration could be linked to the fast replenishment of GSH in our trial. Previously, efficiently reversible GSH depletion following AITC treatment was observed in plant cells *Arabidopsis thaliana* [16] and in yeast *Candida albicans* [28].

Uncontrolled ROS generation impairs endogenous antioxidant defense systems such as dismutase, catalase, and peroxidase superoxide activities [29]. Such enzyme inhibition was also observed in our diabetic rats non-treated with AITC. Conversely, in AITC-treated diabetic rodents, the highest dose of AITC (25 mg/kg b.w.) led to SOD and GPx activity increase. SOD, GPx, and CAT play an essential protective role in maintaining the redox state in mammalian cells [30]. These enzymes counterbalance the toxicity of free radicals. SOD is the first defense line against ROS toxicity. SOD first neutralizes the superoxide anion into the less toxic hydrogen peroxide, which can be further converted into water by GPx and CAT [14,31]. Therefore, the high activity of GPx and CAT is accompanied by a high concentration of H_2_O_2_. Their contribution to hydrogen peroxide detoxication is different because of differences between peroxidase and catalase in the Michaelis constant to H_2_O_2_. CAT plays its role when GPx reaches saturation with the substrate [32]. In the present trial, the highest ROS inducer dose of AITC (25 mg/kg b.w) did not change catalase activity, likely due to an insufficient level of hydrogen peroxide, which did not exceed the GPx saturation. This might be an explanation for why GPx activity was only changed. Previously, downregulation of CAT along with augmented SOD expression was observed by Zhang et al. [15]. In our study, the observed enzyme increase was accompanied by a considerable rise in liver transcription factor NF-E2-related factor 2 (Nrf2) in diabetic rats following 25 mg/kg b.w AITC treatment. Nrf2 is pivotal for protecting cells from oxidative damage [33]. Previously, a significant increase in nuclear Nrf2 gene expression was found in adult albino rats after 25 mg/kg/day for 7 days [24] as well as in C57BL/6 mouse liver after 15 mg/kg b.w./d for 7 days of AITC administered orally via gavage [6]. Moreover, Sahin et al. [34] demonstrated an antioxidant property of 100 mg/kg b.w. per day for AITC administrated orally for 12 weeks to HFD/STZ-induced diabetic rats. Interestingly, AITC intake was characterized by a total antioxidant capacity increase, liver and kidney Nrf2 expression, and MDA decrease. In contrast to Sahin’s data, our research indicated that Nrf2 was noted collaterally with lipid peroxidation caused via AITC presence. Such an increase in nuclear Nrf2 translocation with ROS production by isothiocyanates was previously observed in human adenocarcinoma Caco-2 cells. ITC’s higher concentration was also more efficient [35]. Fourquet and co-workers [36] proved that oxidation of cytosolic protein Keap1 (Nrf2 inhibitor) by oxygen and nitrogen free radicals is important for Nrf2 activation.

A wide range of factors provokes the production of ROS/RNS. These endogenous mediators also include glucocorticoid hormones [37] and cytokines [38]. In our study, increased cortisol concentration by AITC treatment could have raised ROS levels. An inflammatory response also accompanies oxidative stress [39]. Numerous studies indicate ITC’s inflammation modulation ability, mainly due to reduction/inhibition of nuclear factor kappa-light-chain-enhancer of activated B-cells (NF-κB) activity [40]. Recently sinigrin, natural aliphatic glucosinolate, a precursor of allyl isothiocyanate, was shown to inhibit the production of pro-inflammatory cytokines such as tumor necrosis factor-alpha (TNF-α) and interleukin IL-1β, IL-6, and IL-18 through NF-κB inhibition during adipocyte differentiation [41]. Subedi et al. [42] noted that AITC also significantly decreased the expression of NF-κB p65, IL-6; TNF-α in mammary tissues. In addition, 20 mg/kg b.w. AITC showed an anti-inflammatory effect on 7,12-dimethylbenz(a)anthracene (DMBA)- and N-methyl-N-nitrosourea (MNU)-induced mammary carcinogenesis in female Sprague Dawley rats [43]. Very recently, Li Ch-X et al. [44] demonstrated that 4wk oral administration of 100 mg/kg/d AITC substantially decreased the transcription of proinflammatory cytokines TNFα, IL-1β, and IL-6 through attenuation of the nuclear factor kappa B signaling pathway in the liver tissues of HFD-fed mice C57BL/6. Consistent with these in vivo findings, AITC treatment (20 µM/24 h) also significantly decreased TNF and IL-6 mRNA levels in palmitate-acid-treated AML-12 cells [44]. Prawan et al. [13] revealed that several pro-inflammatory mediators and cytokines (iNOS, COX-2, IL-1β, IL-6, and TNF-α) were reduced by ITCs and were related to the downregulation of NF-kappaB signaling pathways in HT-29-N9 human colon cancer cells. Despite this widespread AITC anti-inflammatory evidence, conversely, we noticed its dose-dependent cytokine concentration rise and tendency to proinflammatory upregulation of transcription factor NF-kappaB level in diabetic rats.

It is known that various plant-derived compounds have antioxidant properties via metal-binding abilities. So, they can be used to mitigate oxidative stress and chelate trace elements involved in redox reactions reduce diabetes complications. Many research studies showed a positive association between dietary Fe, Cu intake, and the risk of T2DM. High-fat diet increased hepatic Fe stores in rats. Fe overload can result in glucose intolerance [18]. In our study, we also noticed Fe-elevated content in the diabetic rat liver. In opposition to Fe and Cu, Zn has antioxidant and anti-inflammatory properties. Zn is a cofactor of over 200 key enzymes involved in glucose, lipid, and protein metabolism [20]. According to some reports, Zn can decrease the risk of type 2 diabetes. It is believed to act through the insulin-signaling pathway, and even has insulin-mimetic properties [45]. So far, there is no information about AITC effects on the trace element status in diabetes in the available literature. In this study, we noticed an equivocal influence of AITC on amelioration in mineral homeostasis in the detoxification and lymphoid organs (liver, kidney, spleen). The AITC effect was dose-dependent. Moreover, one dose of AITC (2.5 mg kg b.w.) was able to cause a beneficial decrease in Fe collaterally with an adverse Zn drop in the diabetic rat liver. Hyperglycemia disturbs the proportion of tissular Cu to Zn, leading to increased Cu/Zn ratios, associated with oxidative stress and inflammation. In our trial, a positive effect of lowering the Cu/Zn molar ratio was observed in the spleen, caused by only one dose of AITC (5 mg kg b.w.). Cu and Zn trace elements are also essential in maintaining redox balance as Cu/Zn SOD cofactors. Regarding animal models, Cu-Zn SOD activity was lowered in groups of zinc-deficient rats rather than in rats with proper Zn contents [20]. Despite a drop in the liver Zn amount following 2.5 mg/b.w. AITC treatment, the deficiency of Cu-Zn SOD activity was not observed in our study.

## 4. Materials and Methods

### 4.1. STZ-Induced Experimental Diabetic Rats Fed a High-Fat Diet and Experimental Design

Male Wistar rats (*n* = 48; 8 weeks old, mean bodyweight 180–200 g) were purchased from Breeding Lab Animals (Brwinow, Poland). Throughout the experiment, the rats were kept at an animal care facility under controlled temperature (21 ± 2 C) and humidity (55 ± 5%) with an artificial 12h/12h day/night cycle, with access to food and water ad libitum. After 7 days of adaptation, the animals were divided into two groups: healthy rats (C) (*n* = 8) and type 2 diabetes mellitus rats (T2DM) (*n* = 32). The healthy rat group that served as a control was fed on the regular pellet diet (Labofeed B, Kcynia, Poland). T2DM rats received a high-fat diet (rodent diet with 40% kcal% fat, source; Labofeed B) and water ad libitum.

After six weeks of dietary manipulation, rats from group T2DM were intraperitoneally injected with freshly prepared streptozotocin (STZ) at 35 mg/kg b.w. in an ice-cold citric buffer pH 4.4; 1 mL/kg b.w. The control healthy rat group (C) was injected with the carrier (citric buffer). All animals were further fed proper diets until the end of the experiment. The diabetes induction was confirmed after 72 h of streptozotocin administration by the glucose tolerance test (GTT) and insulin tolerance test (ITT). After confirmation of diabetes, only animals with fasting blood glucose **≥** 250 mg/dL (above 14 mmol/L) were considered diabetic and used for experiments.

### 4.2. AITC Treatment

Allyl isothiocyanate (AITC) was purchased from Sigma-Aldrich (St. Louis, MO, USA; product number 377430; purity 95%). AITC is 3-isothiocyanatoprop-1-ene with the molecular formula CH_2_=CHCH_2_N=C=S; (C_3_H_5_NCS). To explore the effects of AITC in diabetic rats, the animals with induced type 2 diabetes were divided into four experimental groups: T2DM:the high-fat-fed with STZ injection diabetic control group and T2DM + AITC 2.5; 5.0; 25: the high-fat-fed with STZ injection diabetic control groups treated with three different AITC doses, 2.5; 5.0; and 25 mg/kg b.w., respectively. Treatment was administered orally to rats once a day per 14 days, either with deionized water as a vehicle (C and T2DM) or with 2.5, 5, 25 mg/kg b.w. of AITC dissolved in deionized water (T2DM + AITC 2.5; 5.0; 25, respectively) at volume 0.5 mL/100g b.w. AITC was always employed as a freshly prepared solution. Body weight was determined on a two-daily basis.

### 4.3. Data Collection

After 14 days of AITC treatment, the animals were slaughtered after overnight fasting (12 h) before decapitation. Blood samples were obtained by exsanguination. The blood was centrifuged at 3000× *g* for 15 min at 4 °C to obtain the serum. Subsequently, the visceral organs (liver, kidney, and spleen) were excised rapidly, blotted dry, weighed, and washed immediately with normal saline. The obtained blood serum and internal organs were stored at −20 °C until analysis.

### 4.4. Trace Elements Determination in Tissues

The samples of liver, kidney, and spleen were digested in 65% (w/w) spectra pure HNO_3_ (Merck) in the Microwave Digestion System (Speedwave Xpert, Berghof, Germany). After mineralization, Fe, Zn, and Cu concentrations in the mineral solutions were measured by the flame atomic absorption spectrometry method (spectrometer AAS-3, Zeiss, with BC, Germany) [46]. The atomic absorption lines used were Fe—248.3 nm, Zn—213.9 nm, and Cu—324.8 nm. Simultaneous analysis of the certified reference material (bovine liver, SRM 1577C, National Institute of Science and Technology, USA) was undertaken to ensure the accuracy of quantitative determinations of metals. The recoveries of trace elements were as follows: Fe (104%), Zn (95%), and Cu (102%). The content of metals was expressed in µg/g dry mass of tissue.

### 4.5. Determination of Enzyme Activities

Superoxide dismutase (SOD), glutathione peroxidase (GPx), catalase (CAT), and glutathione-S-transferases (GST) activities were measured with rat-specific kits (Cayman Chemical; 1180 East Ellsworth Road, Ann Arbor, MI, USA) in the liver and the blood serum. For determination in the liver, approximately 100 mg of liver tissue was homogenized in 1 mL of the appropriate cold buffer by adding a fresh protease inhibitor cocktail (Complete; Roche Diagnostics; Mannheim, Germany, 1 table per 50 mL). The buffer composition differed depending on the enzymes: 20 mM HEPES buffer, pH 7.2, containing 1 mM EGTA, 210 mM mannitol, and 70 mM sucrose for SOD assay; 50 mM Tris-HCl, pH 7.5, 5mM EDTA, 1mM DTT; 1,4-dithiothreitol for GPx assay; 50 mM potassium phosphate, pH 7.0, containing 1 mM EDTA for CAT assay; 100 mM potassium phosphate, pH 7.0, containing 2 mM EDTA for GST assay. The homogenates were centrifuged at 10,000× *g* for 15 min at 4 °C. The supernatant was removed for the assay and stored on ice. The enzyme activities in the liver were expressed per mg protein. Protein content in homogenates was measured using the bicinchoninic acid (BCA) Kit (Thermo Scientific, Rockford, IL, USA). One unit of SOD was defined as the amount of enzyme needed to exhibit 50% dismutation of the superoxide radical. One unit of GPx was defined as the amount of enzyme that will cause the oxidation of 1.0 nmol of NADPH to NADP per minute at 25 °C. One unit of CAT was defined as the amount of enzyme that will cause the formation of 1.0 nmol of formaldehyde per minute at 25 °C. One GST unit was defined as an enzyme that conjugates 1.0 nmol of CDNB (1-chloro-2,4-dinitrobenzene) with reduced glutathione per minute at 25 °C.

### 4.6. Determination of Proinflammatory Parameters

Cytokines such as IL-1β, IL-6, and TNF–α in the serum were determined with an ELISA kit for the rats (R&D Systems, Inc., 614 McKinley Place NE, Minneapolis, MN 55413, USA). Primary transcription factors: rat nuclear factor NF-kappa-B p105 subunit (NFκβ) and rat nuclear factor erythroid 2-related factor 2 (Nrf2) in the liver and serum and C-reactive protein (CRP) concentrations in the serum were measured with rat-specific kits (EIAab Science Co., Ltd, Biopark, Optics Valley, Wuhan, China, 430074).

### 4.7. Measurements of Cortisol

The cortisol concentration in the serum was determined with the radioimmunoassay method (RIA) using a rat-specific kit (Immunotech, Radiova1; 102 27 Prague 10; Czech Republic).

### 4.8. Measurements of Lipid Peroxidation

The extent of lipid peroxidation in the liver and the blood serum was estimated calorimetrically by measuring thiobarbituric acid reactive substances (TBARS) using a rat-specific assay kit for TBARS (Cayman Chemical, 1180E, Ellsworth Rd, Ann Arbor, MI, USA). Values in the liver and the blood serum were expressed as nmol per g tissue or µmol/L, respectively.

### 4.9. Measurements of the Content of Total, Protein-Bound, and Non-Protein Sulfhydryl Groups

The content of total protein and nonprotein sulfhydryl groups (thiol groups) in the liver was assayed using Ellman’s reagent (5,5-dithiobis-2-nitro benzoic acid as the reagent; DTNB) at 412 nm according to the method described by Sedlak and Lindsay [47].

### 4.10. Statistical Analysis

The results were presented as the arithmetic mean ± SEM. One-way analysis of variance (ANOVA) with post hoc Tukey’s multiple comparison test using the GraphPad Prism for Windows software (license no. GRA/3802/2015, USA) was used to determine statistically significant differences in the means values at *p* ≤ 0.05, *p* ≤ 0.01, and *p* ≤ 0.001. The only differences considered were between C and T2DM and T2DM with 2.5; 5.0; 25 mg/kg b.w. of AITC, respectively.

## 5. Conclusions

This study examined, for the first time, AITC potency in reducing oxidative and inflammatory stress along with its profitable modulation trace element status in rats with diabetes induced by a high-fat diet and streptozotocin. Our findings revealed that two-week AITC treatment enhanced lipid peroxidation and proinflammatory cytokine levels in diabetic rats. Inflammation action of AITC was intensified along with its dose increase. Moreover, AITC showed an ambiguous effect on the mitigation of mineral homeostasis disturbances, which was related to AITC doses and type of organs. These observations do not encourage the use of AITC as a mender of oxidative and inflammation status in diabetes.

## Figures and Tables

**Table 1:** The effect of HFD/STZ injection and AITC administration on mineral homeostasis (Fe, Cu, Zn) in the liver, kidney, and spleen of diabetic rats.

	C	T2DM	T2DM + 2.5 AITC	T2DM + 5 AITC	T2DM + 25 AITC
Liver					
Fe^2+^	373.30 ± 23.66	472.90 ± 20.80 **	400.10 ± 12.06 ^†^	549.50 ± 11.72 ^†^	439.50 ± 12.94
Cu^2+^	8.35 ± 0.61	5.63 ± 0.53 **	4.70 ± 0.32	6.01 ± 0.58	6.15 ± 0.50
Zn^2+^	86.14 ± 6.29	78.08 ± 2.73	64.13 ± 2.71 ^†^	81.35 ± 2.37	85.11 ± 6.27
Cu^2+^/Zn^2+^	0.1126 ± 0.01	0.07457 ± 0.01	0.07336 ± 0.004	0.07422 ± 0.01	0.07957± 0.01
Kidney					
Fe^2+^	220.10 ± 32.95	184.00 ± 12.81	232.20 ± 26.66	238.30 ± 10.03	229.70 ± 36.73
Cu^2+^	26.63 ± 1.69	25.61 ± 2.39	23.26 ± 3.32	20.74 ± 0.88	18.90 ± 1.43
Zn^2+^	99.61 ± 6.53	117.10 ± 6.90	115.20 ± 8.38	95.04 ± 3.17	93.14 ± 4.18
Cu^2+^/Zn^2+^	0.2743 ± 0.02	0.2154 ± 0.01	0.1984 ± 0.02	0.2209 ± 0.01	0.2057 ± 0.02
Spleen					
Fe^2+^	2919 ± 230.50	2161 ± 255.80	2393 ± 400.70	2557± 214.00	2594 ± 368.30
Cu^2+^	6.621 ± 0.47	7.476 ± 0.54	4.929 ± 0.23 ^††^	5.063 ± 0.38 ^†^	7.199 ± 0.72
Zn^2+^	73.75 ± 2.64	75.36 ± 2.95	86.34 ± 7.47	88.95 ± 4.35	84.12 ± 5.97
Cu^2+^/Zn^2+^	0.09004 ± 0.01	0.1013 ± 0.01	0.06034 ± 0.01	0.05649 ± 0.003 ^††^	0.09156 ± 0.01

Data are the mean ± SEM (*n* = 8); ** *p* ≤ 0.01—significant difference between C and T2DM; ^†^ *p*≤ 0.05, ^††^ *p* ≤ 0.01—significant difference between T2DM and T2DM + AITC. C—control group; T2DM—high-fat diet with STZ-injected group; T2DM + AITC—high-fat diet with STZ-injected group with oral administration of 2.5; 5; 25 mg/kg b.w. AITC, respectively.

## Data Availability

Raw data are available from the corresponding author upon reasonable request.

## Sample Availability

Samples of the compounds are not available from the authors.
